# Significant Interactions between Adipokines and Vitamin D Combined with the Estimated Glomerular Filtration Rate: A Geriatric Case Study

**DOI:** 10.3390/jcm12062370

**Published:** 2023-03-19

**Authors:** Monika Biercewicz, Katarzyna Kwiatkowska, Kornelia Kędziora-Kornatowska, Magdalena Krintus, Robert Ślusarz, Barbara Ruszkowska-Ciastek

**Affiliations:** 1 Department of Geriatrics, Faculty of Health Sciences, Collegium Medicum in Bydgoszcz, Nicolaus Copernicus University, M. Skłodowskiej-Curie 9, 85-094 Bydgoszcz, Poland; 2 Department of Pathophysiology, Faculty of Pharmacy, Collegium Medicum in Bydgoszcz, Nicolaus Copernicus University, M. Skłodowskiej-Curie 9, 85-094 Bydgoszcz, Poland; 3 Department of Laboratory Medicine, Faculty of Pharmacy, Collegium Medicum in Bydgoszcz, Nicolaus Copernicus University, M. Skłodowskiej-Curie 9, 85-094 Bydgoszcz, Poland; 4 Neurological and Neurosurgical Nursing Department, Faculty of Health Sciences, Collegium Medicum in Bydgoszcz, Nicolaus Copernicus University, M. Skłodowskiej-Curie 9, 85-094 Bydgoszcz, Poland

**Keywords:** vitamin D, eGFR, adipokines, age-related diseases, geriatric females

## Abstract

Vitamin D deficiency is an important issue in the worldwide population, especially in older people. According to the World Health Organization data, in 2030, 1 in 6 people in the world will be 60 years old or older. The main storage site for vitamin D is adipose tissue. Further, 25(OH)D regulates the expression of adipogenic genes and apoptosis of adipocytes and directly influences the secretion of the appetite-regulating hormone—leptin. Thus, we investigated the impact of the serum concentrations of leptin, adiponectin, omentin, ghrelin, visfatin, and biochemical parameters on vitamin D and estimated glomerular filtration rate (eGFR) in geriatric females. Our studies indicate that the leptin, visfatin and ghrelin are linked with vitamin D concentration and the eGFR rate in the geriatric females. (1) Background: Vitamin D deficiency is common in older people, and researchers are looking for a link between vitamin D deficiency and the occurrence of diseases in advanced age. The study aimed to evaluate the association between serum 25(OH)D levels and clinical variables in older females. (2) Methods: We investigated the impact of the serum concentrations of leptin, adiponectin, omentin, ghrelin, visfatin, and biochemical parameters on vitamin D and estimated the glomerular filtration rate (eGFR) in 74 geriatric females. (3) Results: We observed a significantly higher concentration of creatinine and visfatin in the G2 stage (eGFR = 60–89 mL/min./1.73 m^2^). We performed an additional analysis to exclude the effect of vitamin D supplementation and obtained a significantly higher vitamin D concentration in the G2 stage. We found significantly lower vitamin D concentrations in older people. In addition, in a person with low levels of vitamin D, we observed significantly lower levels of albumin and ghrelin. Older patients (80 to 89 years old) had significantly lower levels of vitamin D, albumin, insulin, HOMA-IR, and ghrelin than younger patients (60 to 69 years old). Spearman’s correlations performed to examine the relationship between clinical variables seemed to confirm previous results. According to ROC curve analysis, leptin concentration was the strongest predictor of vitamin D fluctuations (the area under the curve, AUC = 0.685; with 79.5% sensitivity and 51.4% specificity; *p* = 0.0291). However, visfatin reached the most accurate AUC^ROC^ = 0.651 with 84.2% sensitivity and 49.1% specificity for predicting effects on eGFR. (4) Conclusions: The results suggest that serum levels of leptin, visfatin, and ghrelin are linked with vitamin D concentration and the eGFR rate in the population of geriatric females.

## 1. Introduction

Longevity can be considered a success for mankind. However, the price we have to pay for this is high and associated with the occurrence of many age-related disorders. According to the data from 2020, individuals aged 65 and older represent approximately 20% of the total population in the European Union (EU) [1]. This means that almost one in five people in the EU is aged 65 or over. In the overall U.S. population, people aged 65 and older make up 17% and women almost 18% [2]. According to the World Health Organization data, in 2030, 1 in 6 people in the world will be 60 years old or older [3].

Vitamin D (25(OH)D) deficiency is a serious problem in the worldwide population, especially in older people. To better understand the genesis of vitamin D deficiency, it is necessary to trace the path of vitamin D metabolism. Further, 25(OH)D is supplied to the body in two ways: with food and through skin synthesis. Vitamin D supplied to the body undergoes double hydroxylation—in the liver (hydroxylation at position 25 with the formation of 25-hydroxyvitamin D3 (25OHD); the main metabolite precipitating in the blood) and kidneys (hydroxylation at position 1 with the formation of 1,25-hydroxyvitamin D3 (1,25OHD), a metabolite with hormone activity) [4]. According to current data, hydroxylation at position 1 may also occur in other tissues, e.g., breast, prostate, or macrophages [5].

The role of vitamin D is not limited to its classic function of maintaining calcium and phosphate homeostasis (protective effect against rickets, osteoporosis, and osteomalacia). Vitamin D inhibits activation of the RAS (renin-angiotensin system), which results in decreased nephron destruction and lower blood pressure [6]. It leads to a decrease in endothelin synthesis and vascular smooth muscle cell proliferation and improves vascular endothelial function [7]. In addition, 25(OH)D affects the conversion of proinsulin to insulin and increases tissue sensitivity to insulin [8]. Interactions with the vitamin D receptor (VDR) located on pancreatic beta cells cause insulin secretion [9]. The beneficial effect of vitamin D on immunity is due to the presence of VDR receptors in macrophages. Macrophages recognize bacteria (lipopolysaccharide LPS) through toll-like receptors (TLRs). TLR binding leads to increased expression of both 1-α-hydroxylase and VDR [10]. The neuroprotective effect of vitamin D is related to a reduction of Ca^2+^ level in the brain (high calcium level can cause neurotoxicity), also via inhibition of brain gamma-glutamyl transpeptidase, which reduces hydrogen peroxide concentration by increasing glutathione concentration [11]. Vitamin D demonstrates anti-cancer effects based on mechanisms of proliferation, angiogenesis suppression, and activation of apoptosis. Additionally, 25(OH)D through the VDR binds to a regulatory site in the promoter of the p21 gene, which leads to cell-cycle inhibition in the G1 phase. Vitamin D activates apoptosis by inhibiting the expression of the protooncogene bcl-2 and increasing the expression of the proapoptotic protein Bax. Interestingly, vitamin D inhibits the activation of gene transcription for IL-8, which is a strong stimulant of angiogenesis [12]. Additionally, the main storage site for vitamin D is adipose tissue. Further, 25(OH)D regulates the expression of adipogenic genes and apoptosis of adipocytes and directly influences the secretion of the appetite-regulating hormone—leptin. Some studies indicate another function of vitamin D; it regulates the secretion of adiponectin, which sensitizes tissues to insulin [13].

In the older population, vitamin D deficiency is associated with decreased absorption from the GI tract due to gastroenterological diseases or drugs. In geriatric populations, 25(OH)D deficiency occurs due to the loss of active nephrons. Older individuals avoid exposure to sunlight during summer (a lack of cutaneous synthesis) [5]. Geriatric patients are often malnourished and do not receive adequate supplies of vitamin D from their diet. Some of those patients also experience a loss of body fat and muscle tissue, which causes a change in the secretion of adipokines [14]. Vitamin D deficiency promotes the occurrence of diabetes type 2. Patients have increased secretion of pro-inflammatory cytokines, e.g., IL-6, which is responsible for pancreatic beta cell apoptosis [8,9]. Vitamin D deficiency may lead to neurodegenerative disorders, schizophrenia, and depression. Zdrojewicz et al. have found a correlation between low blood levels of vitamin D and increased incidence and greater aggressiveness of prostate, breast, and colorectal cancers [7].

The main objective of this study was to assess the potential association between serum 25(OH)D levels and clinical variables in the population of geriatric females. Since vitamin D is substantial for numerous physiopathological processes, we also estimated the diagnostic power of selected parameters in the prediction of vitamin D deficiency and kidney function deterioration in older females.

## 2. Materials and Methods

### 2.1. Recruitment and Participants

**Figure 1:**
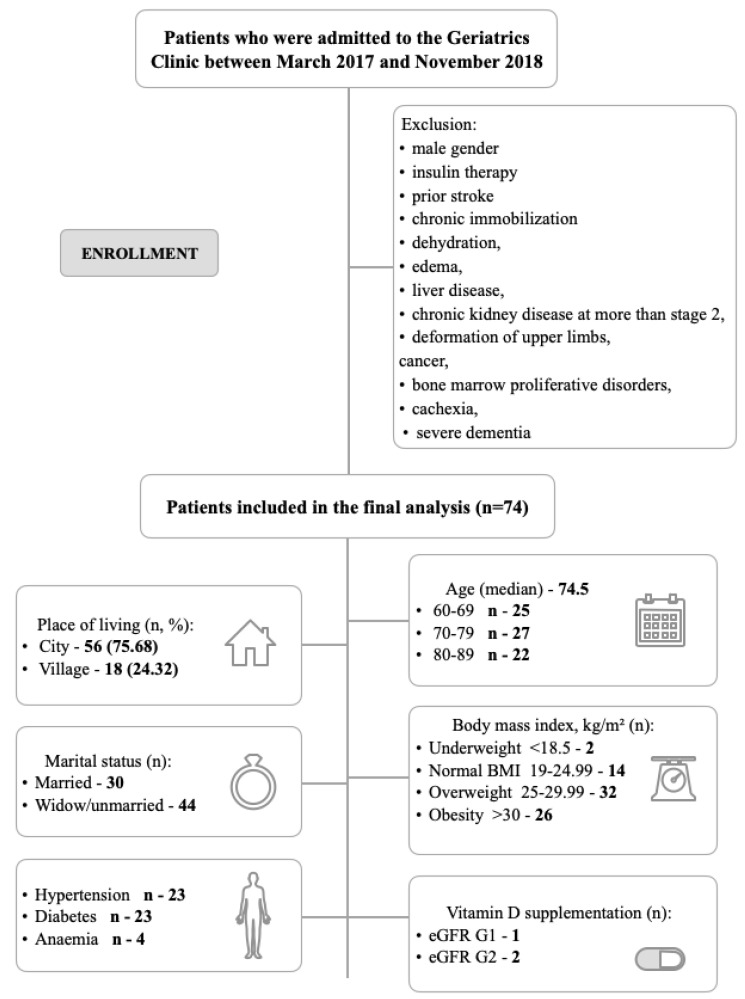
Flowchart of the patient selection, classification, and baseline characteristic.

The study comprised a total of 74 older women. The median age of patients was 74.5 years old (60 to 89 years old). Patients admitted to the Geriatrics Clinic of University Hospital No. 1 in Bydgoszcz, Poland, between March 2017 and November 2018, were selected (Figure 1). Patients were hospitalized to determine the extent of impairment, treatment, rehabilitation priorities, needs, and options for providing further treatment/rehabilitation/care and to determine the elder’s ability to function independently.

The main inclusion criteria were female gender and the age of >60 years. In order to exclude the effect of female hormones, we selected patients who had their last menstruation at the maximum age of 57 years. The exclusion criteria were as follows: dehydration, oedema, liver disease, chronic kidney disease at more than stage 2, deformation of upper limbs, cancer, bone marrow proliferative disorders, cachexia, or severe dementia. The supplementary exclusion criteria were as follows: male gender, chronic immobilization, prior stroke, eGFR (the estimated glomerular filtration rate) < 60 mL/min./1.73 m^2^, and insulin therapy.

### 2.2. Ethical Approval

This trial was performed under appropriate institutional ethics approvals (permission no. KB/470/2016; WN707). Written informed consent was obtained from each patient. This study was performed in accordance with the Helsinki Declaration and relevant regulations.

### 2.3. Demographic Profiles and Clinical Characteristics Collection

Figure 1 and Table 1 show the baseline and clinical characteristics of the enrolled patients. Complete clinical history included information on the participant’s date of birth, residence, marital status, education, smoking habit (current smoker: yes/no), alcohol consumption, day of last menses, number of children, main disease (reason for hospitalization), and other chronic diseases. Most of the women lived in a city (75.68%). The primary diseases that patients suffered from were hypertension (23 patients), diabetes (23 patients), and anaemia (4 patients). Patients were divided into two subgroups according to the eGFR level using recommendations outlined in The Kidney Disease: Improving Global Outcomes (KDIGO). Nineteen of the patients belong to the G1 and 55 to the G2 stage. Only 3 patients out of 74 were supplementing vitamin D (one with the G1 stage and two with the G2). Body mass index (BMI), defined as the body weight (in kilograms) divided by the square of height (in meters), and waist-hip ratio (WHR) were calculated for all patients. Most women, according to the WHO recommendations, were overweight (32 patients) and obese (26 patients).

### 2.4. Biochemical and Hematological Assays

Routine blood tests were performed upon patient admission. The standard blood collection protocols were respected; patients had been fasting, after 30 min of rest and after twelve hours of overnight fasting. Serum parameters, such as vitamin D, albumin, hsCRP (high-sensitivity C-reactive protein), glucose, insulin, creatinine, calcium, and parathyroid hormone (PTH) were determined using automated analyzers applying biochemical methods. Complete blood count was determined by using an automatic white blood cell count.

The blood was collected without anticoagulants, centrifuged, and then stored at –80 °C until analysis.

#### 2.4.1. Serum Leptin Assays

Serum leptin levels were measured using a high-sensitivity human leptin ELISA kit (enzyme-linked immunosorbent assay kit for leptin (LEP) Cloud-Clone Corp., catalogue number SEA084Hu). The detectable range of leptin was 0.156–10 ng/mL. The minimum detection dose of leptin was 0.054 ng/mL, the within-label coefficient of variation was <10% and the run-to-run coefficient of variation was <12%.

#### 2.4.2. Serum Adiponectin Measurement

Serum adiponectin levels were determined using a high-sensitivity human adiponectin ELISA kit (enzyme-linked immunosorbent assay kit for adiponectin (ADPN) Cloud-Clone Corp., catalogue number SEA605Hu). The detection range of adiponectin was 0.156–10 ng/mL. The minimum detectable dose of adiponectin was 0.061 ng/mL, the within-label coefficient of variation (within-run) was <10%, and the between-label coefficient of variation (run-to-run) was <12%.

#### 2.4.3. Serum Omentin Analysis

Serum omentin levels were assayed using a high-sensitivity human omentin ELISA kit (enzyme-linked immunosorbent assay kit for intelectin 1/omentin (ITLN1) Cloud-Clone Corp., catalogue number SEA933Hu). The detectable range of omentin was 1.56–100 ng/mL. The minimum dose of omentin detectable was 0.59 ng/mL, the within-label coefficient of variation (within-run) was <10%, and the between-label coefficient of variation (run-to-run) was <12%.

#### 2.4.4. Serum Ghrelin Assays

Serum ghrelin levels were assessed using a high-sensitivity human ghrelin ELISA kit (enzyme-linked immunosorbent assay kit for ghrelin (GHRL) Cloud-Clone Corp., catalogue number CEA991Hu). The detectable range of ghrelin was 123.5–10,000 pg/mL. The minimum detection dose of ghrelin was 49.5 pg/mL, the intra-label coefficient of variation was <10%, and the inter-run coefficient of variation was <12%.

#### 2.4.5. Serum Visfatin Measurement

Serum visfatin levels were assessed using a high-sensitivity human visfatin ELISA kit (enzyme-linked immunosorbent assay kit for visfatin (VF) Cloud-Clone Corp., catalogue number SEA638Hu). The detection range of visfatin was 1.56–100 ng/mL. The minimum detectable dose of visfatin was 0.55 ng/mL, the intra-series coefficient of variation was <10%, and the inter-series coefficient of variation was <12%.

### 2.5. Anthropometric Calculation

Waist-hip ratio (WHR) was calculated using a computer program. The interpretation of the WHR result is as follows: <0.8 (low), 0.8–0.89 (medium), >0.9 (high).

### 2.6. eGFR Calculation

The estimated glomerular filtration rate (eGFR, (mL/min/per 1.73 m^2^) was estimated according to the 4-variable modification of diet in renal disease (MDRD) study formula as follows:

eGFR = 186 × (serum creatinine (mg/dL))^−1.154^ × (age)^−0.203^ × (0.742 gender index for female)

### 2.7. Statistical/Data Analysis

Statistical analysis was done using Statistica version 13.1 (StatStoft^®^, Cracow, Poland). The Shapiro–Wilk test was used to check the normality of data distribution. Comparisons between two groups of continuous data were performed using the Student t-test (normal distribution) or Mann–Whitney test (non-normal distribution). Comparisons between more than two groups of continuous data were performed by univariate ANOVA analysis with normal distributions or the Kruskal–Wallis ANOVA analysis in the case of variables with non-normal distributions. Patients’ data are presented as mean and standard deviation or median and interquartile range (IQR) as suitable. In order to assess the relations between studied variables, a correlation analysis was performed. Spearman’s rank order correlation test was used to test the correlations between the studied parameters. The receiver operating characteristic curves (ROC), AUC (area under a curve), and Youden’s index were also used in the analysis. Statistical significance was predetermined as *p* < 0.05.

## 3. Results

### 3.1. Baseline Characteristics

Among patients admitted to the Geriatrics Clinic of University Hospital No. 1 in Bydgoszcz, Poland between March 2017 and November 2018, 74 women were selected who passed the inclusion and exclusion criteria for the study. Median age was 74.5 age (IQR 60–89 years old). Twenty-five women were between 60–69 years, 70–79 years were 27 women, and 22 women were between 80–89 years. According to The Kidney Disease: Improving Global Outcomes (KDIGO) recommendation, 19 of patients belong to G1 and 55 to G2 stage. In agreement with the body mass index (BMI), most women were overweight (32 patients) and obese (26 patients). Table 1 and Table 2 present the characteristics of the study population.

### 3.2. eGFR Assessment

Table 3 presents results for the clinical variables classified by eGFR stages. According to KDIGO classes, patients were divided into two stages: 19 cases were G1 and 55 subjects were G2. Significant higher concentrations of creatinine and visfatin were observed in the G2 stage (*p* ≤0.0001, *p* = 0.0521 respectively). Analysis showed a significant upward trend toward higher vitamin D levels in G2 stage subjects (*p* = 0.0757).

Additional analysis was performed in order to exclude the effect of vitamin D supplementation. Three patients were excluded from the analysis: one with G1 stage and two with G2 stage. According to this analysis, significant difference was reached with respect to vitamin D concentration and CKD stages (*p* = 0.0466, median G1 = 13.65 ng/mL, G2 = 18.70 ng/mL), (Table S1. Clinical characteristics of patients according to KDIGO classes excluding vitamin D supplementation).

### 3.3. Association between Vitamin D Concentration and Clinical Variables

Table 4 shows the differences between clinical characteristics according to vitamin D level. Patients were divided into three equal groups. Lower levels of vitamin D were observed in older individuals (*p* = 0.0001). It was observed that older people with relevant vitamin D deficiency (<15 mg/dL) showed significantly lower albumin and ghrelin levels (*p* = 0.0015, *p* = 0.0397, respectively). Additionally, the concentrations of hsCRP, creatinine, and leptin tend to be significant; hsCRP and leptin have higher levels in those patients who had a lower concentration of vitamin D (*p* = 0.0954, *p* = 0.0575, respectively). The concentration of creatinine has decreasing levels when there are lower levels of vitamin D (*p* = 0.0911).

### 3.4. Age Groups Analysis

Table 5 shows the comparison of clinical variables according to the age of the patients. Participants of this study were divided into three age subgroups: 60 to 69 years old (25 women), 70 to 79 years old (27 women), and 80 to 89 years old (22 women). Older patients (80 to 89 years) showed significantly lower levels of vitamin D, albumin, insulin, HOMA-IR, and ghrelin than younger patients (60 to 69 years old) (*p* = 0.0082, *p* = 0.0004, *p* = 0.0208, *p* = 0.0079, *p* = 0.0086, respectively). However, there was a trend toward lower glucose levels in older people than in younger individuals (*p* = 0.0677). The concentration of adiponectin was higher in older patients compared to those who were younger (*p* = 0.0627).

### 3.5. Relationship between Clinical Variables

**Figure 2:**
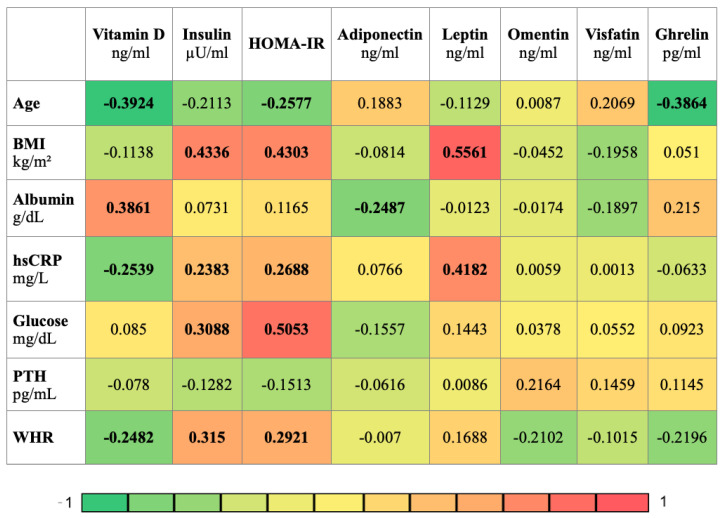
Heatmap displaying the r values obtained from Spearman correlation analysis performed among clinical variables; *p*-values < 0.05 were considered to indicate statistical significance and are marked in bold. BMI, body mass index; hs-CRP, high sensitivity C-reactive protein; PTH, parathyroid hormone; WHR, waist-hip ratio; HOMA-IR, Homeostatic Model Assessment—Insulin Resistance.

**Figure 3:**
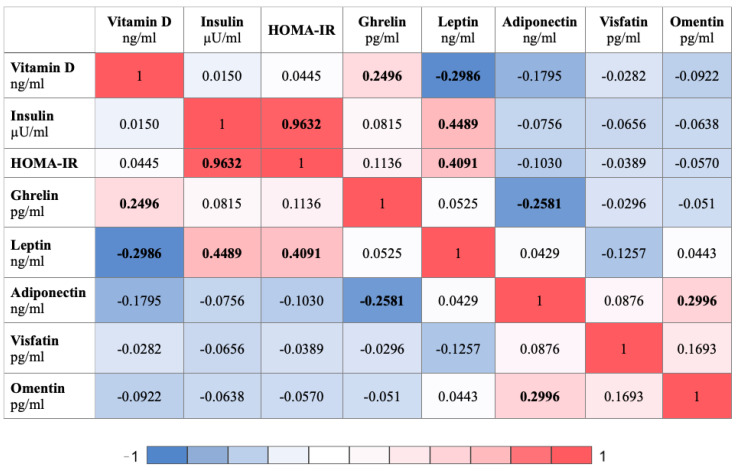
Heatmap displaying the r values obtained from Spearman correlation analysis performed among clinical variables; *p*-values < 0.05 were considered to indicate statistical significance and are marked in bold. HOMA-IR, Homeostatic Model Assessment—Insulin Resistance.

Correlation analysis was performed to find relationships between the clinical variables. The analysis was carried out using Spearman’s rank correlation and is presented in the form of heatmaps (Figure 2 and Figure 3). 

As a result (Figure 2), vitamin D was found to correlate negatively with age, hsCRP, WHR (r = −0.3924, r = −0.2539, r = −0.2482) and correlate positively with albumin (r = 0.3861). Insulin positively correlated with hsCRP, glucose, WHR, (r = 0.2382, r = 0.3088, r = 0.315) and most strongly with BMI (r = 0.4336). HOMA-IR negatively correlated with age (r = −0.2577) and positively correlated with BMI, hsCRP, glucose, WHR (r = 0.4303, r = 0.2688, r = 0.5053, r = 0.2921). Adiponectin negatively correlated with albumin (r = −0.2487). Leptin positively correlated with BMI, hsCRP (r = 0.5561, r = 0.4182). Ghrelin negatively correlated with age (r = −0.3864).

In Figure 3, vitamin D positively correlated with ghrelin (r = 0.2496) and negatively correlated with leptin (r = −0.2986). Insulin positively correlated with HOMA-IR and leptin (r = 0.9632, r = 0.4489). HOMA-IR positively correlated with leptin (r = 0.4091). Ghrelin negatively correlated with adiponectin (r = −0.2581). Adiponectin positively correlated with omentin (r = 0.2996).

### 3.6. Tests of Sensitivity and Specificity

**Figure 4:**
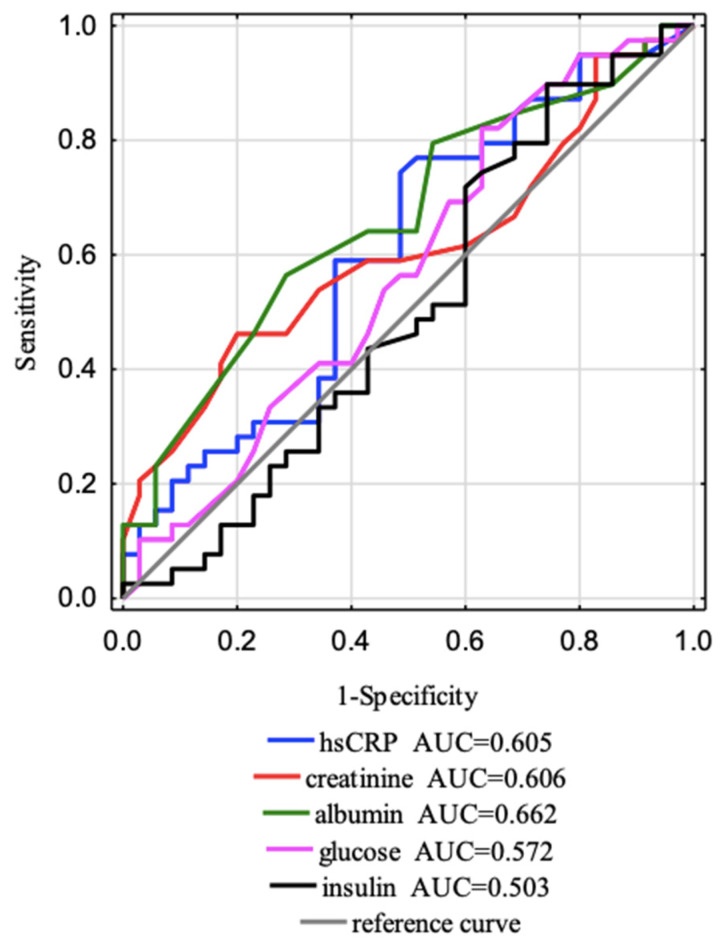
Five ROC curves and AUC values of the clinical parameters studied were designed to distinguish factors that affect vitamin D concentration (cut-off point 18.5 ng/mL).

**Figure 5:**
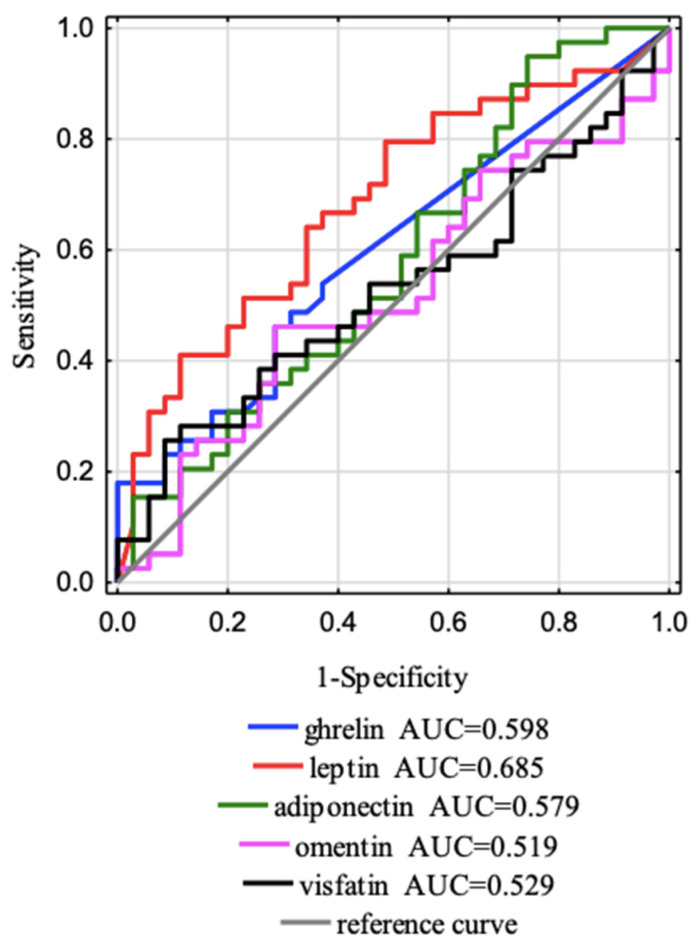
Five ROC curves and AUC values of the adipokines studied were designed to distinguish factors that affect vitamin D concentration (cut-off point 18.5 ng/mL).

The ROC (receiver operating characteristic) curves for separate laboratory parameters were constructed to distinguish factors affecting vitamin D concentrations (Table 6, Figure 4 and Figure 5). The areas under the curve with a 95% confidence interval were established (AUC, 95% thresholds with sensitivity and specificity). The borderline of the diagnostic usefulness of the test based on the AUC > 0.5 and *p* < 0.05 was reached for albumin and leptin. We found out that the cut-off point for albumin was 3.70 g/dL with 56.4% sensitivity and 71.4% specificity; for leptin, it was 7.38 ng/mL with 79.5% sensitivity and 51.4% specificity. The most accurate AUCROC value for predicting effects on vitamin D is the AUC for leptin which is 0.685.

**Figure 6:**
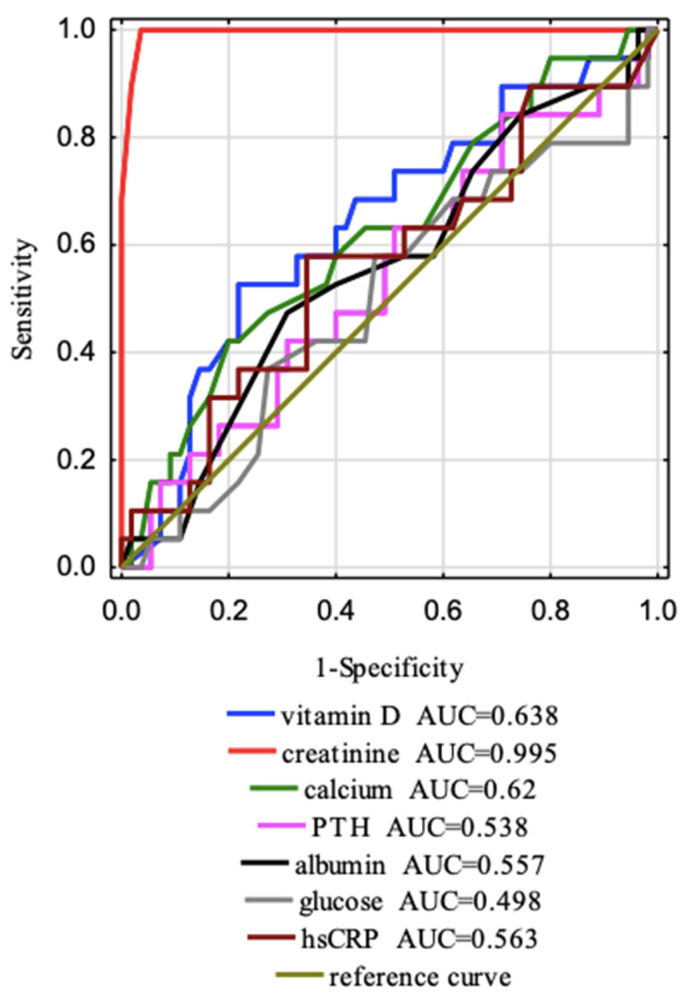
Seven ROC curves and AUC values of the clinical parameters were designed to distinguish factors that affect the eGFR stages (G1 and G2).

**Figure 7:**
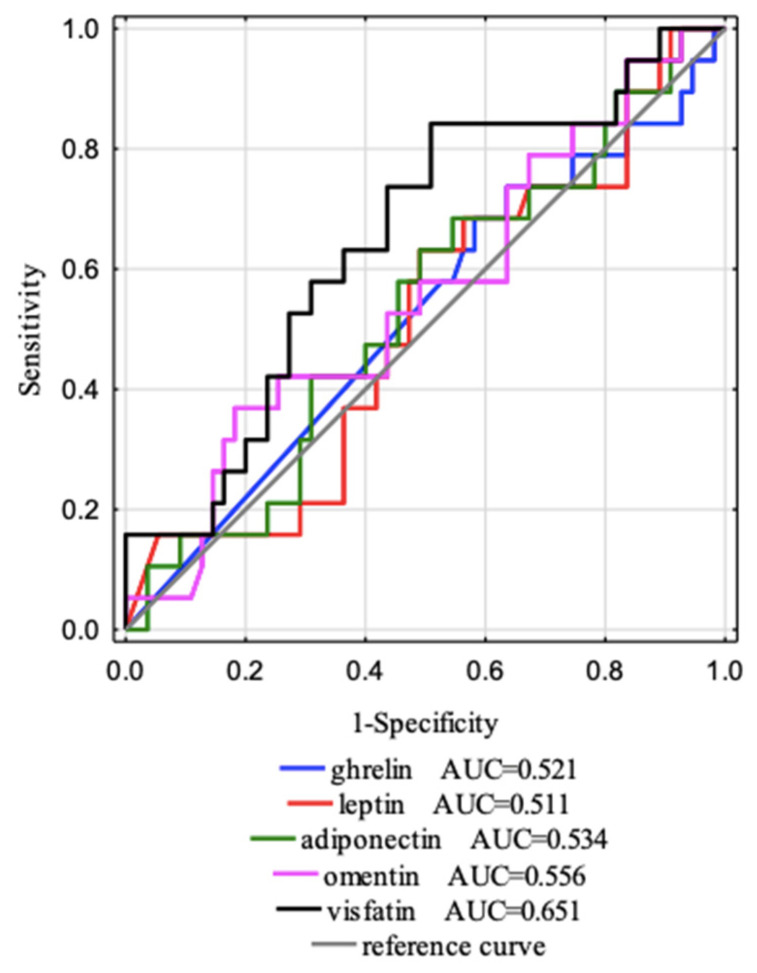
Five ROC curves and AUC values of the adipokines studied were designed to distinguish factors that affect the eGFR stages (G1 and G2).

The ROC curves (Figure 6 and Figure 7) for separate laboratory parameters were constructed to distinguish factors that affect the eGFR stages (G1 and G2). All data are presented in Table 7. The areas under the curve with a 95% confidence interval were established (AUC, 95% thresholds with sensitivity and specificity). The borderline of the diagnostic usefulness of the test based on the AUC > 0.5 and *p* < 0.05 was reached for creatinine and was 0.68 mg/dL with 100.0% sensitivity and 96.4% specificity and for visfatin was 22,450.00 ng/mL with 84.2% sensitivity and 49.1% specificity. Additionally, we observed a tendency that vitamin D affects eGFR with a cut-off point of 13.9 mg/dL with 52.5% sensitivity and 78.2% specificity. The most accurate AUC^ROC^ value for predicting effects on eGFR is the AUC for creatinine (0.995), which is obvious because eGFR is calculated from creatinine. The following AUC was for visfatin (AUC = 0.651).

## 4. Discussion

Physiological aging is a process of progressive, regressive, and irreversible changes in the tissues and organs of the body, determined by genetic factors and modified by coexisting diseases, lifestyle, and environmental factors. At the biological level, physiological aging leads to physicochemical changes in cells, including degeneration, apoptosis, amyloid accumulation, metabolism slowdown, and the impaired ability for self-regulation, adaptation, and regeneration. With the discovery of receptors for vitamin D located in many different tissues and organs, the role of vitamin D and its effect on body function have become the focus of numerous investigations. Epidemiological studies show a link between vitamin D deficiency and the incidence of diseases of old age [15]. Adipose tissue, the largest organ in humans, is associated with longevity mechanisms and metabolic disorders of old age. With age, the distribution and composition of adipose tissue, and the profile of secreted adipokines change and the glomerular filtration rate declines [16,17].

In the first stage of our study, we compared patients with different eGFR stages (G1 and G2) and various clinical parameters. Paradoxically, in our analysis, we observed that there is a significantly higher concentration of vitamin D in the G2 stage than in the G1 stage (after the elimination of supplementary impact). Our results from ROC curves demonstrated that vitamin D effects on the eGFR stages have a tendency toward significance. Visfatin is a protein, which is one of the adipokines. Its most important action is to act as a proinflammatory cytokine, which stimulates the expression of inflammatory cytokines, such as interleukin 6 (IL-6), tumor necrosis factor α and β. The study of Syed Ali Fathima et. al. suggested that visfatin can be a novel marker of endothelial dysfunction in CKD patients [18]. This is confirmed by our research in which we observed a significantly higher concentration of visfatin in the G2 stage than G1. Furthermore, the ROC curve indicated that visfatin affects an eGFR stage (AUC = 0.651) and the cut-off was 22450.00 ng/mL with 84.2% sensitivity and 49.1% specificity. Based on this, it is possible to distinguish between patients with the G1 and G2 stages of CKD.

The study group was divided into three almost equal groups according to vitamin D concentrations. In our analysis, we observed that vitamin D concentrations were lower in older patients and those with lower albumin levels. A study by Kwon et al. showed that concomitant reductions in vitamin D and albumin in older people are associated with reduced muscle strength and balance ability, which may translate into a loss of independence [19]. These results are confirmed by Spearman’s correlation, in which vitamin D correlates negatively with age. Vitamin D is absorbed from the intestine and the skin. This may be due to avoidance of sun and gastrointestinal problems [4]. Additionally, the concentration of vitamin D depends on the dietary intake; unfortunately, geriatric patients often develop nutritional disorders, and patients are often malnourished and experiencing the ‘anorexia of aging’ [20]. Ghrelin is a peptide hormone that plays a key role in the neurohormonal regulation of food intake (stimulates appetite), energy homeostasis, and growth hormone (GH) secretion [21]. In our study, along with a lower concentration of vitamin D, we also observed a lower concentration of ghrelin. These results are confirmed by Spearman’s correlation, in which vitamin D correlates positively with ghrelin. Vitamin D plays a role in stimulating the secretion of adipokines (leptin, adiponectin, resistin) and consequently affects the energy homeostasis of the body [13]. Our results suggest that vitamin D also affects ghrelin secretion. Furthermore, in our study, we observed higher leptin levels in patients with low vitamin D levels (with a trend toward significance). These results are confirmed by Spearman’s correlation, in which vitamin D correlates negatively with leptin. Our results from ROC curves demonstrated that leptin has a major effect on vitamin D concentration (AUC = 0.685), and the cut-off for leptin was 7.38 ng/mL with 79.5% sensitivity and 51.4% specificity. Based on this, it is possible to distinguish between patients with low and high concentrations of vitamin D (cut-off point 18.5 ng/mL). Leptin is a peptide hormone synthesized by white adipose tissue [22]. It regulates energy homeostasis, reproductive and immune function, and metabolism (satiety hormone) [23]. Our results confirm the researchers’ findings. Gangloff et. al. showed an inverse relationship between vitamin D concentration and leptin secretion [24]. In addition, in our study, we observed higher C-reactive protein levels in patients with low vitamin D levels (with a trend toward significance). These results are confirmed by Spearman’s correlation, in which vitamin D correlates negatively with hsCRP. Laird et al. suggested that there is an inverse correlation between vitamin D levels and CRP levels as a biomarker of inflammation [25]. Vitamin D plays a key role in the modulation of the inflammatory system, regulates the production of inflammatory cytokines, and inhibits the proliferation of pro-inflammatory cells [26]. Vitamin D deficiency, through impaired cellular response, affects the immune system, which is related to the occurrence of, for example, inflammatory bowel diseases. Vitamin D modulating the immune system can also affect the course of cancer and infectious diseases [27].

The study cohort was categorized into three subgroups: 60 to 69 years (25 women), 70 to 79 years (27 women), and 80 to 89 years (22 women). In our study, we observed that the concentration of vitamin D was lower in older patients. Aging plays an important role in the conversion of active forms of vitamin D. Several studies suggested that the production of active forms of vitamin D is reduced by 50% as a result of a decline in functioning nephrons. Vitamin D concentrations are also affected by exposure to sunlight. From April to September, the concentration of this vitamin increases by 10 ng/mL, resulting in only 4 months of the year where the concentration of the vitamin is lower. However, this is not always the case for older people, as they often avoid sunlight [28]. Decreased albumin levels as a result of malnutrition are the strongest predictor of morbidity and mortality. In our study, we observed a lower concentration of albumin in older patients. Vitamin D modulates pro-inflammatory cytokines that are associated with malnutrition, i.e., IL-1, Il-7, TNF-α [29]. The phenomenon of glucose intolerance associated with aging is not entirely clear. Glucose intolerance is a component of many factors, including increased body fat mass, decreased physical activity, medications, concomitant diseases, defects in insulin secretion, and decreased liver sensitivity that inhibit glucose output [30]. In our study, we observed a decrease in insulin concentration in relation to age, which supports the thesis that insulin secretion is impaired in older patients. Our observation is consistent with Gary et al. who observed that insulin decreases with age as a result of progressive damage to organs such as the pancreas [31]. Our results are of interest because we observed significantly lower concentrations of glucose due to age (with a tendency to significance). Based on the data obtained, it can be speculated that hyperglycemia may be observed less frequently in aging women. Homeostasis model assessment of insulin resistance (HOMA-IR) is a simple and useful method for evaluating insulin sensitivity. In our study, HOMA-IR reduced with age. These results are confirmed by Spearman’s correlation, in which HOMA-IR correlates negatively with age. Ghrelin stimulates appetite and the secretion of growth hormone (GH). In our study, we observed that ghrelin secretion was lower in older patients. These results are confirmed by Spearman’s correlation, in which HOMA-IR correlates negatively with ghrelin. Decreased GH secretion is associated with decreased lean body mass and increased fat mass. This altered lipid metabolism results in increased mortality (development of vascular disease) [32]. One of the factors affecting ghrelin secretion is sex hormones. In this study, they were eliminated because the patients were postmenopausal. Adiponectin is a protein synthesized in adipose tissue. It reduces insulin resistance and has anti-inflammatory and anti-atherosclerotic effects. Serum adiponectin levels are affected by many factors, including age [33]. In our study, we observed higher concentrations of adiponectin in patients aged 70 to 79 than in those aged 60 to 69 years (with a tendency to significance). Considering that adiponectin has an anti-atherosclerotic effect, it is interesting to note that it increases in older people, which may suggest that older people are somehow not likely to develop atherosclerosis.

### Limitations of the Study

There are some limitations that we would like to introduce. We enrolled a small number of patients; which could constitute a bias; data should be confirmed in larger populations. The study was performed in a daily clinical routine, the sample size was dependent on receiving patients’ consent for participation and meeting with patients’ restricted inclusion criteria. Since we only analyzed women of Polish descent, our findings are, therefore, not necessarily directly suitable for other ethnic groups

## 5. Conclusions

Despite the limited number of geriatric individuals included in the study, our results uncover some relevant issues: (1) The link between the concentration of vitamin D and eGFR was found in geriatric populations. Based on our study, we postulate that vitamin D concentration can predict chronic kidney disease. (2) According to the Youden index, we suggest that the cut-off point 22,450.00 ng/mL of visfatin concentration assessed in serum through an immunoenzymatic method may serve as a value which discriminates between patients with impaired eGFR and those without it. (3) Our results indicate that leptin may be used as an adequate, non-invasive prognostic biomarker of vitamin D deficiency in older females. Leptin reached the most accurate AUC^ROC^ = 0.685; (*p* = 0.0291) for vitamin D deficiency prediction, with a cut-off value of 7.38 ng/mL with 79.5% sensitivity and 51.4% specificity. (4) Lower vitamin D concentration is associated with lower ghrelin levels in older women and indirectly with nutritional status expressed by low albumin levels. (5) Further study in this regard is needed to confirm our findings.

## Figures and Tables

**Table 1:** Biochemical and anthropometric characteristics of participants.

Variable (Units)	Participants (N = 74)	Reference Values *
Vitamin D (ng/mL)	18.51 ± 7.77	30.0–100.0
Creatinine (mg/dL)	0.76 ± 0.10	0.5–0.8
eGFR (mL/min/1.73 m^2^)	80.08 ± 11.37	>60.0
Calcium (mmol/L)	2.28 ± 0.11	2.1–2.7
Parathyroid hormone (pg/mL)	64.33 ± 33.70	18.5–88.0
Albumin (g/dL)	3.86 ± 0.35	4.02–4.76
Glucose (mg/dL)	96.01 ± 26.24	70.0–99.0
Insulin (µU/mL)	10.04 ± 9.94	3.0–25.0
HOMA-IR	2.56 ± 3.19	<2 (normal)
hsCRP (mg/L)	3.13 ± 4.47	0–5.0
Leptin (ng/mL)	8.64 ± 5.01	0.156–10.0
Adiponectin (ng/mL)	24,112.81 ± 4839.17	0.156–10.0
Omentin (ng/mL)	108.83 ± 54.21	1.56–100.0
Ghrelin (pg/mL)	9602.92 ± 643.43	123.5–10,000.0
Visfatin (ng/mL)	24,393.77 ± 19,059.31	1.56–100.0
Body mass index (kg/m^2^)	28.59 ± 5.30	19–24.99 (normal)
WHR	0.89 ± 0.07	<0.8 (low)

Data are presented as means ± standard deviation. PTH, parathyroid hormone; HOMA-IR, Homeostatic Model Assessment—Insulin Resistance; hsCRP, high-sensitivity C-reactive protein; LAR, leptin/adiponectin ratio; WHR, waist-hip ratio. * Reference values according to the University Hospital No. 1 Laboratory in Bydgoszcz.

**Table 2:** Chronic kidney disease staging recommendation based on KDIGO.

Glomerular Filtration Rate Category	Estimated Glomerular Filtration Rate Range (mL/min/1.73 m^2^)	Severity Description
G1	≥90	Normal or high
G2	60–89	Mildly Decreased
G3a	45–59	Mildly to Moderately Decreased
G3b	30–44	Moderately to Severely Decreased
G4	15–29	Severely Decreased
G5	<15	Kidney Failure

**Table 3:** Patients’ clinical characteristics according to KDIGO classes.

Variable/ Unit	eGFR		
	G1 *n* = 19	G2 *n* = 55	*p*-Value
Age	74.79 (7.74)	74.42 (7.20)	0.8497
BMI kg/m^2^	28.20 25.30/33.70	28.10 25.50/31.20	0.6119
Vitamin D ng/mL	13.90 11.00/20.90	18.90 14.50/24.30	0.0757
Creatinine mg/dL	0.65 0.63/0.67	0.80 0.75/0.85	**<0.0001**
Calcium mmol/L	2.23 2.18/2.31	2.28 2.22/2.33	0.1229
PTH pg/mL	60.60 38.30/77.70	62.20 39.40/80.90	0.6294
Albumin g/dL	3.82 (0.36)	3.87 (0.35)	0.5866
Glucose mg/L	88.00 84.00/100.00	89.00 83.00/100.00	0.9852
HOMA-IR	2.04 1.37/3.34	1.62 1.03/2.10	0.1311
hsCRP mg/L	1.98 0.87/4.53	1.39 0.78/3.28	0.4211
Leptin ng/mL	9.68 5.36/13.39	10.70 2.75/13.12	0.8917
Adiponectin ng/mL	24,790.00 21,165.00/26,050.00	24,340.00 21,920.00/26,575.00	0.6650
Omentin ng/mL	101.70 66.79/122.30	104.60 77.28/136.00	0.4691
Ghrelin pg/mL	1000.00 9433.00/1000.00	1000.00 9379.00/1000.00	0.7771
Visfatin ng/mL	13,510.00 10,575.00/22,310.00	22,270.00 12,675.00/30,605.00	**0.0521**
WHR	0.91 (0.06)	0.88 (0.08)	0.0905

Data are presented as mean ± standard deviation or median and inter-quartile range; BMI, body mass index; PTH, parathyroid hormone; hsCRP, high-sensitivity C-reactive protein; HOMA-IR, Homeostatic Model Assessment—Insulin Resistance; WHR, waist-hip ratio, bold *p*-values denote significant differences.

**Table 4:** Patients’ clinical characteristics according to vitamin D levels.

Variable/ Unit	Vitamin D (Concentration ng/mL)						
	>21 *n* = 24	15–21 *n* = 25	<15.00 *n* = 25	*p*-Value	I vs. II *p*-Value	I vs. III *p*-Value	II vs. III *p*-Value
Age	71.29 (6.26)	72.92 (6.42)	79.20 (6.85)	**0.0001**	0.6584	**0.0003**	**0.0032**
BMI kg/m^2^	28.61 (6.27)	27.82 (4.31)	29.33 (5.31)	0.6078	0.8622	0.885	0.5787
hsCRP mg/L	1.20 0.51/3.28	1.35 0.78/2.95	1.98 1.07/5.06	0.0954	1.0000	0.1227	0.3046
Creatinine mg/dL	0.79 (0.08)	0.77 (0.09)	0.73 (0.11)	0.0911	0.7033	0.079	0.3425
eGFR (mL/min/1.73 m^2^)	77.25 (9.34)	79.60 (10.61)	83.28 (13.33)	0.1735	0.7458	0.1530	0.4826
Albumin g/dL	3.99 (0.33)	3.94 (0.34)	3.66 (0.30)	**0.0015**	0.8465	**0.0024**	**0.0109**
Glucose mg/dL	90.00 84.00/104.50	90.00 84.00/93.00	87.00 84.00/94.00	0.6410	1.0000	1.0000	1.0000
Insulin mU/mL	8.05 5.05/12.90	7.20 5.60/10.50	7.30 5.80/9.50	0.8951	1.0000	1.0000	1.0000
HOMA-IR	1.89 1.16/3.11	1.67 1.27/2.34	1.62 1.22/2.07	0.7637	1.0000	1.0000	1.0000
Ghrelin pg/mL	10,000.00 9758.00/10,000.00	10,000.00 9626.00/10,000.00	9748.00 9012.00/10,000.00	**0.0397**	1.0000	0.0698	0.3390
Leptin ng/mL	9.17 2.24/12.17	8.22 2.76/12.56	12.32 7.71/14.15	**0.0575**	1.0000	0.1148	0.1187
Adiponectin ng/mL	23,982.50 19,090.00/25,622.50	24,790.00 23,110.00/26,185.00	24,825.00 22,175.00/28,355.00	0.1979	0.6362	0.2392	1.0000
Omentin ng/mL	94.79 75.60/125.80	107.50 79.97/122.30	104.60 74.90/151.90	0.8935	1.0000	1.0000	1.0000
Visfatin ng/mL	17,855.00 11,730.00/25,270.00	20,815.00 15,535.00/31,080.00	19,540.00 9660.00/26,950.00	0.4132	0.6718	1.0000	0.8558
WHR	0.87 (0.07)	0.88 (0.08)	0.91 (0.07)	0.1279	0.8795	0.1281	0.2977

Data are presented as mean ± standard deviation or median and inter-quartile range; BMI, body mass index; hsCRP, high-sensitivity C-reactive protein; HOMA-IR, Homeostatic Model Assessment—Insulin Resistance; WHR, waist-hip ratio; bold *p*-values denote significant differences.

**Table 5:** Patients’ clinical characteristics according to age.

Variable/ Unit	Age						
	60–69 *n* = 25	70–79 *n* = 27	80–89 *n* = 22	*p*-Value	I vs. II *p*-Value	I vs. III *p*-Value	II vs. III *p*-Value
BMI kg/m^2^	29.00 25.70/32.80	27.80 25.30/32.00	27.35 22.00/31.20	0.3653	1.0000	0.487	1.0000
hsCRP mg/L	1.35 0.87/3.33	1.47 0.78/3.41	1.49 1.03/4.37	0.9085	1.0000	1.0000	1.0000
Vitamin D ng/mL	20.90 15.70.24.50	19.20 14.70/22.40	13.20 8.80/18.10	**0.0082**	1.0000	**0.0081**	0.0686
Creatinine mg/dL	0.78 (0.10)	0.76 (0.08)	0.75 (0.11)	0.5374	0.9169	0.5335	0.7636
eGFR (mL/min/1.73 m^2^)	80.24 (10.82)	79.33 (10.37)	80.82 (13.44)	0.9009	0.9566	0.9839	0.8950
Albumin g/dL	4.05 (0.36)	3.85 (0.32)	3.65 (0.28)	**0.0004**	0.0855	**0.0005**	0.1108
Glucose mg/dL	91.00 85.00/105.00	92.00 85.00/100.00	85.00 82.00/89.00	0.0677	1.0000	0.1378	0.1148
Insulin mU/mL	7.80 6.20/10.70	8.20 5.80/14.50	6.35 4.40/7.80	**0.0208**	1.0000	0.0638	**0.0315**
HOMA-IR	2.04 1.39/2.40	1.84 1.37/3.72	1.35 0.97/1.66	**0.0079**	1.0000	**0.0202**	**0.0178**
Ghrelin pg/mL	10,000.00 10,000.00/10,000.00	9959.00 9214.00/10,000.00	9727.00 8394.00/10,000.00	**0.0086**	0.2077	**0.0161**	0.8437
Leptin ng/mL	12.56 5.23/13.36	10.48 2.76/12.56	8.21 2.75/12.32	0.4296	1.0000	0.5979	1.0000
Adiponectin ng/mL	23,675.00 19,515.00/25,145.00	25,170.00 23,750.00/26,755.00	24,187.00 21,165.00/27,380.00	0.0627	0.0582	0.3989	1.0000
Omentin ng/mL	103.30 79.47/124.00	101.70 69.41/115.40	111.05 59.22/154.50	0.4153	0.7743	1.0000	0.7653
Visfatin ng/mL	19,520.00 8955.00/24,655.00	20,625.00 12,519.00/29,225.00	21050.00 11,100.00/41,675.00	0.3391	0.7445	0.52	1.0000
WHR	0.89 (0.06)	0.89 (0.09)	0.89 (0.07)	0.9933	0.9929	0.9984	0.9986

Data are presented as mean ± standard deviation or median and inter-quartile range; BMI, body mass index; HOMA-IR, Homeostatic Model Assessment—Insulin Resistance; hsCRP, high sensitivity C-reactive protein, WHR, waist-hip ratio; bold *p*-values denote significant differences.

**Table 6:** Results of diagnostic accuracy for the prediction of vitamin D fluctuation.

ROC Data	Stimulant	Destimulant	Destimulant	Destimulant	Stimulant	Destimulant	Stimulant	Stimulant	Destimulant	Destimulant
	hsCRP mg/L	Creatinine mg/dL	Albumin g/dL	Glucose mg/dL	Insulin mU/mL	Ghrelin pg/mL	Leptin ng/mL	Adiponectin ng/mL	Omentin ng/mL	Visfatin ng/mL
AUC	0.605	0.606	0.662	0.572	0.503	0.598	0.685	0.579	0.519	0.529
Youden index	0.26	0.26	0.28	0.19	0.15	0.18	0.31	0.21	0.18	0.17
Cut-off point	1.06	0.72	3.70	97.0	4.70	8394.00	7.38	19,670.00	88.13	9660.00
Sensitivity (%)	74.4	46.2	56.4	82.1	89.7	17.9	79.5	94.9	46.2	25.6
Specificity (%)	51.4	80.0	71.4	37.1	25.7	100	51.4	25.7	71.4	91.4
Positive predictive value (%)	63.0	72.0	68.8	59.3	57.4	100	64.6	58.7	64.3	76.9
Negative predictive value (%)	64.3	57.1	59.5	65.0	69.2	52.2	69.2	81.8	54.3	52.5
Accuracy (%)	63.5	62.2	63.5	60.8	59.5	56.8	66.2	62.2	58.1	56.8
*p*-value	0.1140	0.1079	0.0103	0.2877	0.9620	0.1374	0.0291	0.2407	0.7754	0.6731

**Table 7:** Results of diagnostic accuracy for prediction of eGFR variability.

ROC Data	Destimulant	Destimulant	Destimulant	Destimulant	Destimulant	Destimulant	Stimulant	Stimulant	Destimulant	Stimulant	Destimulant	Destimulant
	Vitamin D ng/mL	Creatinine mg/dL	Calcium mmol/L	PTH pg/mL	Albumin g/dL	Glucose mg/dL	hsCRP mg/L	Ghrelin pg/mL	Leptin ng/mL	Adiponectin ng/mL	Omentin ng/mL	Visfatin ng/mL
AUC	0.638	0.995	0.62	0.538	0.557	0.498	0.563	0.521	0.511	0.534	0.556	0.651
Youden index	0.31	0.96	0.22	0.13	0.16	0.11	0.23	0.10	0.14	0.14	0.19	0.33
Cut-off point	13.90	0.68	2.20	78.20	3.60	88.00	1.91	9820.00	10.55	24,385.00	74.01	22,450.00
Sensitivity (%)	52.6	100.00	42.1	84.2	47.4	57.9	57.9	68.4	63.2	63.2	36.8	84.2
Specificity (%)	78.2	96.4	80.0	29.1	69.1	52.7	65.5	41.8	50.9	50.9	81.8	49.1
Positive predictive value (%)	45.5	90.5	42.1	29.1	34.6	29.7	36.7	28.9	30.8	30.8	41.2	36.4
Negative predictive value (%)	82.7	100	80.0	84.2	79.2	78.4	81.8	79.3	80.0	80.0	78.9	90.0
Accuracy (%)	71.6	97.3	70.3	43.5	63.5	54.1	63.5	48.6	54.1	54.1	70.3	58.1
*p*-value	0.0668	<0.0001	0.1134	0.6285	0.4608	0.9807	0.4298	0.7932	0.8857	0.6556	0.4637	0.0370

## Data Availability

The data presented in this study are available in this article.

## Supplementary Materials

The following supporting information can be downloaded at: https://www.mdpi.com/article/10.3390/jcm12062370/s1, Table S1: Clinical characteristics of patients according to KDIGO classes excluding vitamin D supplementation and Table S2: Patients’ clinical characteristics according to vitamin D levels (median distribution).
