# Supplementary material for: Significant Interactions between Adipokines and Vitamin D Combined with the Estimated Glomerular Filtration Rate: A Geriatric Case Study
Source: J Clin Med. 2023 Mar 19;12(6):2370. doi: 10.3390/jcm12062370 (PMC10052050; doi:10.3390/jcm12062370)
Supplement: Supplementary file 1 [file jcm-12-02370-s001.zip › jcm-2263435-supplementary.pdf]

SUPPLEMENTARY MATERIAL

Table S1 shows the results for clinical variables classified by eGFR grade excluding the effect of vitamin D supplementation. Three patients were excluded from the analysis, one with G1 stage and two with G2 stage. The significant higher concentration of vitamin D and creatinine were observed in G2 stage (P = 0.0466, P < 0.0001, respectively).

Table S1. Clinical characteristics of patients according to KDIGO classes excluding vitamin D supplementation.

Variable/ Unit	eGFR		
	**G1** n=18	**G2** n=53	***P*-value**
**Age**	75.11 (7.83)	74.28 (7.29)	0.6688
**BMI** kg/m²	28.60 25.30/33.70	28.20 25.50/31.20	0.5172
**Vitamin D** ng/ml	13.65 11.00/19.00	18.70 14.50/23.10	**0.0466**
**Creatinine** mg/dl	0.65 0.63/0.67	0.80 0.76/0.85	**<0.0001**
**Calcium** mmol/l	2.25 (0.09)	2.29 (0.11)	0.3144
**PTH** pg/mL	64.21 (37.51)	66.02 (32.88)	0.4575
**Albumin** g/dl	3.82 (0.37)	3.87 (0.36)	0.7554

**Glucose**	88.00	89.00	0.9736
mg/L	84.00/100.00	83.00/100.00	
**HOMA-IR**	2.11	1.67	0.2021
	1.37/3.34	1.11/2.10	
**hsCRP**	1.99	1.40	0.2963
mg/L	0.95/4.53	0.94/3.28	
**Leptin**	9.94	10.91	0.9473
ng/mL	6.37/13.39	2.76/13.12	
**Adiponectin**	24745.00	24324.00	0.6389
ng/mL	21165.00/26030.00	21920.00/26185.00	
**Omentin**	102.70	103.30	0.5698
ng/mL	66.79/122.30	77.28/124.00	
**Ghrelin**	10000.00	10000.00	0.7447
pg/mL	9433.00/10000.00	9443.00/10000.00	
**Visfatin**	14912.50	20815.00	0.0945
ng/mL	10575.00/22310.00	12675.00/29225.00	
**WHR**	0.91	0.88	0.3106
	(0.06)	(0.08)	

Data are presented as mean ± standard deviation or median and inter-quartile range; BMI, body mass index; hs-CRP, high-sensitivity C-reactive protein; HOMA-IR, Homeostatic Model Assessment - Insulin Resistance; WHR, waist-hip ratio; bold p-values denote significant differences.

Table S2 shows the differences between clinical characteristics according to vitamin D level. Patients were divided according to the median into 2 subgroups. We observed significantly lower concentration of vitamin D (P = 0.0008) in older individuals. In patients with lower concentration of vitamin D we observed significantly lower levels of albumin

(P= 0.0162) and significantly higher levels of leptin (P= 0.0064). The concentration of creatinine has decreasing potential according to lower levels of vitamin D (P = 0.0669).

Table S2. Patients' clinical characteristics according to vitamin D levels (median distribution).

Variable/ Unit	Vitamin D (concentration ng/ml)		
	**<18.5** n = 39	**>18.5** n = 35	**P-value**
**Age**	77.13 (6.90)	71.60 (6.65)	**0.0008**
**BMI** kg/m²	28.66 (4.67)	28.51 (6.00)	0.9075
**hsCRP** mg/L	1.67 1.04/4.39	1.04 0.65/3.28	0.1215
**Creatinine** mg/dl	0.74 (0.10)	0.78 (0.09)	0.0669
**eGFR** (mL/min/ 1.73 m²)	81.95 (12.52)	78.00 (9.68)	0.1367
**Albumin** g/dl	3.77 (0.35)	3.96 (0.33)	**0.0162**
**Glucose** mg/dl	88.00 83.00/94.00	90.00 84.00/105.00	0.2906
**Insulin** mU/mL	7.30 5.80/10.40	7.80 4.50/11.50	0.9655
**HOMA-IR**	1.62 1.22/2.40	1.83 1.12/2.50	0.7576

**Ghrelin** pg/mL	9903.00<br>9085.00/10000.00	10000.00<br>9452.00/10000.00	0.1166
**Leptin** ng/mL	12.29<br>7.47/13.52	6.37<br>2.18/11.66	**0.0064**
**Adiponectin** ng/mL	24700.00<br>22225.00/26720.00	24520.00<br>19515.00/26050.00	0.2467
**Omentin** pg/mL	104.60<br>69.12/124.00	103.30<br>76.88/136.00	0.7783
**Visfatin** pg/mL	19540.00<br>9660.00/29785.00	20170.00<br>12675.00/29225.00	0.6768
**WHR**	0.90<br>(0.08)	0.87<br>(0.06)	**0.0418**

Data are presented as mean ± standard deviation or median and inter-quartile range; BMI, body mass index; hs-CRP, high-sensitivity C-reactive protein; HOMA-IR, Homeostatic Model Assessment - Insulin Resistance; WHR, waist-hip ratio; bold p-values denote significant differences.
